# The Common *PKD1* p.(Ile3167Phe) Variant Is Hypomorphic and Associated with Very Early Onset, Biallelic Polycystic Kidney Disease

**DOI:** 10.1155/2023/5597005

**Published:** 2023-07-28

**Authors:** Miranda Durkie, Christopher M. Watson, Peter Winship, Anne-Cecile Hogg, Rodney Nyanhete, Sharon Cooley, Manoj K. Valluru, Charles Shaw-Smith, Coralie Bingham, Mark Gilchrist, Janna Kenny, Genomics England Research Consortium, Albert C. M. Ong

**Affiliations:** ^1^Sheffield Diagnostics Genetic Service, North East and Yorkshire Genomic Laboratory Hub, Sheffield Children's NHS Foundation Trust, Sheffield, UK; ^2^Leeds Genetics Laboratory, North East and Yorkshire Genomic Laboratory Hub, St. James's University Hospital, Leeds, UK; ^3^Leeds Institute of Medical Research, University of Leeds, St. James's University Hospital, Leeds, UK; ^4^Obstetrics and Gynaecology, Rotunda Hospital, Dublin, Ireland; ^5^Kidney Genetics Group, Academic Nephrology Unit, Department of Infection, Immunity and Cardiovascular Disease, University of Sheffield Medical School, Sheffield, UK; ^6^Clinical Genetics, Royal Devon University Healthcare NHS Foundation Trust, Gladstone Road, Exeter, UK; ^7^University of Exeter Medical School, Royal Devon University Healthcare NHS Foundation Trust, Barrack Road, Exeter EX2 5DW, UK; ^8^Clinical Genetics, Children's Health Ireland, Crumlin, Ireland; ^9^Genomics England Research Consortium, UK

## Abstract

Biallelic *PKD1* variants, including hypomorphic variants, can cause very early onset polycystic kidney disease (VEO-PKD). A family with unexplained recurrent VEO-PKD and neonatal demise in one dizygotic twin was referred for clinical testing. Further individuals with the putative hypomorphic *PKD1* variant, p.(Ile3167Phe), were identified from the UK 100,000 genomes project (100 K), UK Biobank (UKBB), and a review of the literature. We identified a likely pathogenic *PKD1* missense paternal variant and the putative hypomorphic *PKD1* variant from the unaffected mother in the deceased twin but only the paternal *PKD1* variant in the surviving dizygotic twin. Analysis of 100 K cases identified a second family with two siblings with similar biallelic inheritance who presented at birth with VEO-PKD and reached kidney failure in their teens unlike other affected relatives. Finally, a survey of 618 UKBB cases confirmed that adult patients monoallelic for *PKD1* p.(Ile3167Phe) had normal kidney function. Our data reveals that p.(Ile3167Phe) is the second most common *PKD1* hypomorphic variant identified and is neutral in heterozygosity but is associated with VEO-PKD when inherited *in trans* with a pathogenic *PKD1* variant. Care should be taken to ensure that it is not automatically filtered from sequence data for VEO cases.

## 1. Introduction

Autosomal dominant polycystic kidney disease (ADPKD), due to pathogenic variants in *PKD1* or *PKD2* genes, is generally an adult-onset disorder which commonly progresses to kidney failure. Very rarely, the presentation can be very early onset (VEO), presenting *in utero* or the neonatal period with a severe phenotype occasionally leading to neonatal demise.

Several case reports [1–3] and two recent series of VEO cases [4, 5] have elucidated the underlying genetic mechanism of disease. In most cases, biallelic *PKD1* variants are detected, more rarely biallelic *PKD2* variants [5, 6] and very rarely trans-heterozygous variants in *PKD1/PKD2/PKHD1/HNF1B* [5, 7]. Fully penetrant pathogenic biallelic *PKD1* variants are assumed to be early gestational embryonic lethal. Hence, all VEO cases reported have at least one variant with likely partial protein function, also known as a hypomorphic variant. In such cases, the hypomorphic variant does not cause ADPKD in isolation in heterozygous parents. The variant classification for hypomorphic variants is, however, problematic as these alleles may be present at high frequency in population studies, are likely to be benign when monoallelic, and could be automatically filtered from sequence data [5].

## 2. Materials and Methods

### 2.1. VEO-PKD Pedigree

A family with a history of VEO-PKD and neonatal demise was referred for NHS diagnostic testing. The couple's first pregnancy was a male foetus with enlarged cystic kidneys and anhydramnios, detected antenatally at 21 weeks. The neonate (II.1) was delivered at 33 weeks gestation but suffered neonatal demise shortly after birth. Array CGH and genetic testing for autosomal recessive polycystic kidney disease (ARPKD) did not identify a molecular diagnosis and DNA was not stored for further testing.

The couple's subsequent pregnancy was dizygotic twins. At 16 weeks gestation, the male foetus II.2 had enlarged bright cystic kidneys detected by ultrasound. In contrast, his twin sister (II.3) had normal kidney echogenicity and length at 16 weeks. At 32 weeks, II.2 had anhydramnios with polycystic kidneys measuring >99.6^th^ centile; II.3 had a few kidney cysts detectable antenatally, with normal kidney length. Both foetuses were delivered at 33 + 5 weeks gestation. II.2 subsequently died shortly after birth, but II.3 has remained clinically well postnatally. There was no known significant family history at the time of referral. The paternal grandfather had a kidney removed for an unknown indication, and the paternal mother had died from cancer aged 41.

A DNA sample extracted from an uncultured postmortem skin sample from II.2 underwent next-generation sequencing (NGS) and dosage analysis using a custom hybridisation capture 17-gene cystic disease diagnostic panel sequenced on a HiSeq 2000 [5]. Dosage analysis for whole exon deletions and duplications was performed using comparative depth of coverage of NGS data (DeCON software ([8]; local validation data for single and multiexon CNVs sensitivity > 0.999 and specificity 0.989).

### 2.2. Genomics England 100,000 Genomes Project

Two separate searches of the 100 K results were undertaken: (1) individuals where the *PKD1* gene was analysed in the selected virtual clinical panel and (2) all samples where the *PKD1* gene had not been analysed as part of their clinical analysis. For full details, see supplementary methods.

### 2.3. MinION Long-Read Sequencing to Determine Phase

To determine the phase of the variants in 100 K cases 1a and 1b, a 7.5 kb long-range PCR product, including exons 15-33, was sequenced using a Flongle flowcell on a MinION long-read sequencer (Oxford Nanopore Technologies (ONT), Oxford, UK). Full details are reported in the supplementary methods.

### 2.4. UK Biobank

The UKBB comprises approximately 500,000 participants with extensive phenotyping and genetic data linked to clinical care records [9]. We examined the UKBB cohort exome data for the presence of the *c*.9499*A* > *T* p.(Ile3167Phe) variant in the *PKD1* gene and obtained demographic data from baseline assessment including age and sex. Clinically relevant phenotype data including CKD-EPI eGFR, systolic and diastolic blood pressure, and ACR from enrolment in UKBB were obtained along with HES data for CKD and cysts. Statistical differences in clinical data were determined by independent *t*-test for continuous data.

## 3. Results

### 3.1. VEO-PKD Neonate Had Biallelic PKD1 Variants including p.(Ile3167Phe)

NGS sequence and dosage analysis in neonate II.2 identified a *PKD1* (NM_001009944.3) *c*.2534 *T* > *C* p.(Leu845Ser) likely pathogenic missense variant and several *PKD1* variants of uncertain significance (*c*.4681*C* > *T* p.(Pro1561Ser), *c*.9499*A* > *T* p.(Ile3167Phe), and *c*.11957*C* > *T* p.(Ala3986Val)). Familial testing showed that the *c*.2534 *T* > *C* p.(Leu845Ser) likely pathogenic missense variant and the *c*.4681*C* > *T* p.(Pro1561Ser) variant of uncertain significance were inherited from the proband's father. A diagnostic kidney ultrasound on the asymptomatic father (age 31) detected multiple small bilateral renal cysts with normal-sized kidneys (9 cm), suggestive of early-stage ADPKD.

The *c*.9499*A* > *T* p.(Ile3167Phe) and *c*.11957*C* > *T* p.(Ala3986Val) variants were shown to be inherited from the unaffected mother. Obstetric kidney ultrasound scans on the mother (age 29) did not detect any kidney cysts. Based on familial testing, the variant classification (https://www.acgs.uk.com/media/11631/uk-practice-guidelines-for-variant-classification-v4-01-2020.pdf) was revised and two variants were classified as unlikely to be clinically relevant (Supplementary Table 1).

**Figure 1:**
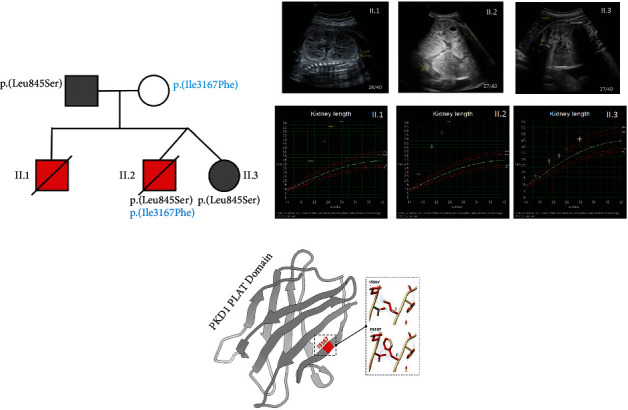
Details of the clinical VEO-PKD pedigree. (a). Family tree showing known *PKD1* genotypes with the two neonates with VEO-PKD and early demise (red). (b). Antenatal scans (top) of all 3 affected neonates at different stages of gestation: II:1 (32 weeks), II:2 (27 weeks); II:3 (27 weeks). Sequential kidney lengths (bottom) for all 3 plotted relative to the 95^th^ centiles for age. (c). *In silico* modelling of the PC1 PLAT domain by missense 3D showing the position of the hypomorphic change p.(Ile3167Phe) using 1 letter amino acid code (I3617F) due to space constraints.

Testing of the clinically well twin sister II.3 confirmed that she had inherited the *c*.2534 *T* > *C* p.(Leu845Ser) likely pathogenic missense variant from her father but not the *c*.9499*A* > *T* p.(Ile3167Phe) variant from her mother. Pedigree, ultrasound scans, and antenatal kidney length from II.1, II.2, and II.3 are shown in Figure 1.

### 3.2. Individuals with the p.(Ile3167Phe) Variant in 100 K

#### 3.2.1. Pedigree 1

Interrogation of the 100 K data for individuals where the applied clinically relevant panel included *PKD1*, identified 16 individuals heterozygous for the p.(Ile3167Phe) variant. Thirteen cases did not have any relevant HPO terms such as cystic kidneys or enlarged kidneys. However, three patients from two families, with HPO terms for cystic kidney disease were found to be heterozygous for this variant. Interestingly, all 3 individuals were also heterozygous for *PKD1* truncating variants and all had severe PKD with early kidney failure. No other likely causative variants were detected in the Cystic kidney disease virtual panel (Genomics England PanelApp) (https://panelapp.genomicsengland.co.uk/panels/283/).

**Figure 2:**
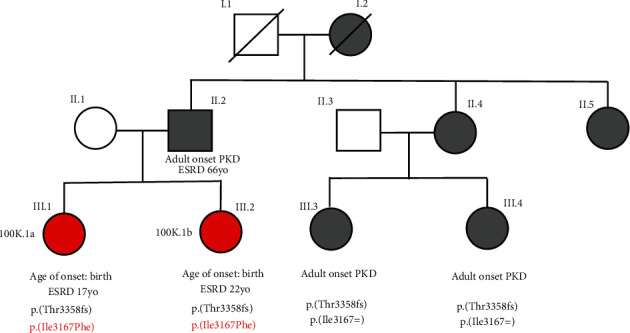
Details of the 100 K.1 pedigree. Family tree showing the two individuals with VEO-PKD (red) and biallelic *PKD1* variants p.(Thr3358Hisfs^∗^32) and p.(Ile3167Phe) who reached kidney failure aged 17 and 22, respectively, within the context of other known affected individuals in the pedigree with only the p.(Thr3358Hisfs^∗^32) variant and/or typical adult-onset PKD.

Proband 1 (100 K.1a) and her affected sibling (100 K.1b) were heterozygous for the c.10071dup p.(Thr3358Hisfs^∗^32) pathogenic variant and the p.(Ile3167Phe) variant. Both had presented at birth with bilateral renal cysts, enlarged kidneys, and hypertension. Their kidney function was noted to be mildly reduced at ages 3 and 5, respectively, but the exact values were not recorded. Both siblings required dialysis and transplantation aged 17 and 22, respectively. There was a paternal history of ADPKD, although of a very different severity, with the affected father starting dialysis followed by kidney transplantation aged 66. DNA sequence analysis in 2 affected paternal cousins identified the c.10071dup p.(Thr3358Hisfs^∗^32) pathogenic variant with no evidence of the p.(Ile3167Phe) variant; therefore, the variants are highly likely to be *in trans* (Figure 2). Nonetheless, since no DNA was available from the affected father, the rare possibility that the p.(Ile3167Phe) variant was a *de novo* event on the same allele as c.10071dup p.(Thr3358Hisfs^∗^32) could not be excluded.

MinION nanopore long-read sequencing was performed on 100 K.1a and 1b to confirm phase (Supplementary Table 2). Parental haplotypes based on the reference/nonreference nucleotide at position chr16:2100465 (c.9499) were established based on the proportion of reads at the downstream duplication site (chr16:2097964, c.10071) that contained an insertion was recorded (Supplementary Figure 1). While the identification of insertion-deletion variants from long-read nanopore datasets remains challenging, our data is consistent with the *c*.9499*A* > *T* p.(Ile3167Phe) and c.10071dup variants being arranged in *trans*, i.e., on different parental haplotypes. The presence of ~6% of c.9499 T (chr16:2100465A) reads containing apparent insertions is likely due to a combination of (i) chimeric read formation (strand switching) during long-range PCR enrichment of the target locus and (ii) a reduction in base-calling accuracy caused by the duplicated base occurring at the end of a string of four G nucleotides.

#### 3.2.2. Pedigree 2

100 K.2 proband was heterozygous for *c*.1198*C* > *T*, p.(Arg400Ter) pathogenic variant and the p.(Ile3167Phe) variant. Clinical details from her clinician stated very severe PKD at referral with an eGFR of 22 ml/min/1.73m^2^, diagnosis at age 12, and approaching kidney failure at age 32. There was, however, insufficient information on her relatives outside the UK, and no DNA was available to confirm the phase.

#### 3.2.3. Other Individuals with the p.(Ile3167Phe) Variant in 100 K

A second search of the 100 K data, where the *PKD1* gene was not included in the relevant clinical panel analysis, identified a total of 99 unaffected heterozygotes and 1 homozygote. The homozygote was recruited as the unaffected parent of a child with multiple congenital anomalies including macrocephaly, hearing impairment, and dysmorphism but with no kidney disease. The unaffected homozygote, aged 49, has no recorded history of renal disease, and a kidney ultrasound scan did not identify any cysts (personal communication).

### 3.3. Phenotypic Data on Individuals with the p.(Ile3167Phe) Variant in UKBB

A total of 643 heterozygotes with the p.(Ile3167Phe) variant were identified from UKBB. Phenotypic data from 618 heterozygote patients of White European descent were compared to 450,375 controls well matched for ethnicity, age, and sex (Supplementary Table 3). There was no evidence of chronic kidney disease (CKD) in the heterozygotes based on baseline measurements of eGFR, BP, and ACR. However, two individuals had a diagnosis of polycystic kidneys, unspecified on HES data. Both were diagnosed in the seventh decade of life or later. Neither individual had CKD based on GFR criteria alone. Without information on family history or imaging, it was not possible to determine whether this represents a true diagnosis of ADPKD or an incidental finding of acquired cysts.

## 4. Discussion

The major finding of this paper is genetic evidence that the common *c*.9499*A* > *T* p.(Ile3167Phe) *PKD1* variant manifests as a hypomorphic variant. Data from gnomAD v2.2.1 (https://gnomad.broadinstitute.org/variant/16-2150466-T-A?dataset=gnomad_r2_1) shows the *c*.9499*A* > *T* variant is present in 340/280802 alleles from multiple ethnicities, including 2 homozygotes, with the highest allele frequency of 0.21% in the European Non-Finnish population. It is found on 181/152192 alleles including 1 homozygote on gnomAD 3.1.1 (https://gnomad.broadinstitute.org/variant/16-2100465-T-A?dataset=gnomad_r3) with the highest allele frequency 0.23% (NFE). This variant fulfils the ACMG criteria BS1 (allele frequency is greater than expected for the disorder) and BS2 (observed in a healthy adult individual for a recessive (homozygous), dominant (heterozygous), or X-linked (hemizygous) disorder with full penetrance expected at an early age) and, therefore, could be classified as class 1 benign without further investigation. Based on this high population frequency, it is likely to be automatically filtered by bioinformatic pipelines designed to select rare variants for analysis. It may also be filtered from WES or WGS trio analysis where the inheritance pattern is selected as autosomal dominant or complete penetrance where it has been inherited from an unaffected parent, or it could be manually discarded by analysts without specialist knowledge of VEO-PKD and hypomorphic variants.

The most commonly reported hypomorphic variant identified in more than ten published VEO-PKD families is p.(Arg3277Cys) [2–5, 10], present on 44 alleles and no homozygotes in gnomAD. *PKD1* p.(Arg3277Cys) is the only *PKD1* hypomorphic variant with experimentally proven reduced function. Studies using an engineered mouse model showed it to be a temperature-sensitive folding/trafficking mutant with approximately 20% retained activity in a compound heterozygous Arg3277Cys/null mouse mutant, consistent with a hypomorphic effect [11]. *PKD1* p.(Arg3277Cys) homozygotes have been reported to develop adult-onset PKD with kidney failure or transplantation at ages 62, 75, and 50 consistent with this dosage-dependent model [2, 4]. Based on functional studies, we would assume homozygotes have ~40% functional polycystin-1 (PC1) protein. However, since the reported homozygotes do not have a more severe presentation than classic ADPKD with a heterozygous null allele, the level of functional PC1 protein may be closer to 50% [12].

**Figure 3:**
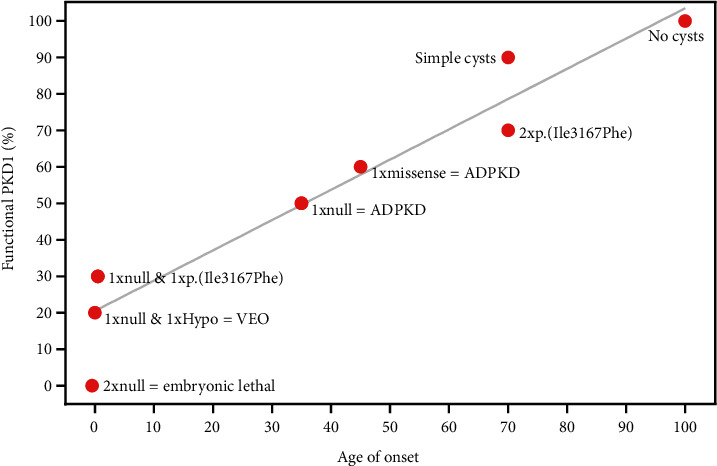
Dosage model of cyst formation. *PKD1* dosage (%, *y*-axis) is shown to vary from 0 (2 null alleles) to 100 (2 normal alleles) and the likely age of presentation for different variants shown on the *x*-axis (years). The total dosage corresponding to the inheritance of each variant as monoallelic (1 normal and 1 variant allele) or biallelic (2 variant alleles) is illustrated. We predict that p.(Ile3167Phe) must have ~30% retained PC1 activity since p.(Ile3167Phe) homozygotes have been reported without kidney failure. The cystogenic threshold is assumed to be <30% functional PC1.

For p.(Ile3167Phe), two homozygotes have been reported in gnomAD (1 exome aged 55-60, 1 genome aged 40-45) although their phenotype is unknown. A search of 100 K genomes and UK Biobank identified a single p.(Ile3167Phe) homozygous individual who was recruited as the parent of a child affected with multiple congenital anomalies but with no HPO terms for cystic kidneys or kidney disease. Kidney ultrasound did not detect any cysts in this individual at the age of 49 years though no kidney function tests were available. Assuming this is correct, the absence of cysts in this homozygote could imply that the retained activity of PC1 in the p.(Ile3167Phe) homozygote is sufficient to avoid cystogenesis, i.e., >55-60%. This would imply that a compound heterozygote for a null allele and p.(Ile3167Phe) has approximately 30% retained PC1 activity (Figure 3). Unlike p.(Arg3277Cys), however, our phenotypic information is currently limited to a single individual. In addition, more sensitive kidney imaging (e.g., MR or CT) in more individuals will be needed to exclude the presence of tiny microscopic cysts below the sensitivity of ultrasound detection.

There is currently no direct functional evidence of the pathogenicity of *PKD1* p.(Ile3167Phe). At codon 3167, both isoleucine and valine amino acids are commonly found in mammals, fish, and reptiles, and this region of the signature PLAT domain is conserved down to *Cioana intestinalis*. *In silico* tools have provided inconclusive data. The p.Ile3167Phe variant has an intermediate predicted pathogenicity score of 0.459 from REVEL, likely due to the presence of Ile and Val. The Ile→Phe variant is predicted as deleterious/damaging by Provean/SIFT, respectively, and probably damaging by PolyPhen-2 HumDiv and HumVar. Ile3167 is located in the PLAT (polycystin-1, lipoxygenase, and alpha toxin) domain, which regulates PC1 trafficking to the plasma membrane. The structure is a *β*-sandwich, with four strands in each sheet. The p.Ser3164 residue is critical for phosphorylation and function of this domain, and several likely pathogenic variants are reported in this region including the neighbouring 3168 amino acids at codon 3168 (p.(Arg3162Leu), p.(Phe3168Leu), p.(Phe3168del), and p.(Ala3171Pro)) (https://pkdb.mayo.edu/variants). Interestingly, p.(Arg3162Cys) has previously been reported as a hypomorphic variant, inherited *in trans* with a pathogenic truncating *HNF1β* mutation in a child with early-onset ADPKD and normal parents [7]. Functional studies showed loss of *in vitro* Ser3164 phosphorylation and reduction of surface delivery to the plasma membrane and primary cilia predicted to be due to protein misfolding and impaired surface delivery by approximately 30%–50% [13]. *In silico* protein modelling by Missense3D for the p.(Ile3167Phe) missense variant predicts that it could affect a phosphatidylserine (PS) binding pocket in PLAT and, therefore, affect membrane association (Figure 1(c)) [5, 13].

We previously reported the p.(Ile3167Phe) variant in two VEO-PKD neonates with severe disease [5]. Two additional VEO-PKD families (one with 2 affected siblings) have since been reported in the literature, both with p.(Ile3167Phe) inherited from the unaffected parent and a truncating variant inherited from the affected parent [10, 14]. All 10 cases from 7 families are summarised in Table 1. Notably, while all cases show paediatric onset and a severe phenotype compared to classic adult-onset ADPKD, there is a significant variation in disease severity with cases ranging from neonatal demise to kidney failure aged 32. This variation could be due to unknown *in cis* or *in trans* genetic modifiers.

Variant classification for hypomorphic variants is challenging with no guidelines currently in place and several ACMG codes not being applicable or difficult to apply [5]. Not surprisingly, p.(Ile3167Phe) has been reported on ClinVar as likely benign (×2) or as a variant of uncertain significance (VUS × 4) (ClinVar variant ID: 440135). Guidelines for low penetrance and risk alleles using a modified version of the ACMG classes recommend the terms uncertain risk allele, likely risk allele, and established risk allele depending on the number of established case-control studies or a meta-analysis confirming a significant odds ratio [15]. Functional studies are not required for established risk alleles due to the small effect that may not be detectable in many assays. We propose the use of equivalent terminology for hypomorphic variants: uncertain hypomorphic variant, likely hypomorphic variant, and established hypomorphic variants depending on the number of reported cases and weight of evidence. Case-control studies for rare hypomorphic variants are not feasible. Functional studies may be challenging for some hypomorphic variants depending on the sensitivity of the assay to detect partial defects. Based on this terminology, we suggest that p.(Arg3277Cys) is an established hypomorphic variant, and p.(Ile3167Phe) is a likely hypomorphic variant.

*PKD1* p.(Ile3167Phe) is now the second most common hypomorphic variant reported in VEO-PKD. Based on gnomAD frequency alone, it is likely to be excluded from analysis by automated variant interpretation pipelines. Care should be taken to ensure this variant is analysed in all VEO cases due to the high recurrence risk in the affected families.

## Figures and Tables

**Table 1:** Summary of the VEO-PKD cases from our study and the literature.

Case	Presentation	Variant 1 (V1)	Parental origin V1	Variant 2 (V2)	ACMG classification V2	Parental origin V2	Reference
1	Neonatal	*c*.9499*A* > *T* p.(Ile3167Phe)	Maternal (unaffected)	c.10326_10356del p.(Gly3443Serfs^∗^20)	Pathogenic	*de novo*	[5]
2	Neonatal	*c*.9499*A* > *T* p.(Ile3167Phe)	Maternal (unaffected)	c.755del p.(Pro252Argfs^∗^38)	Pathogenic	Paternal (affected)	[5]
3a & b	Antenatal (TOP x2)	*c*.9499*A* > *T* p.(Ile3167Phe)	Maternal (unaffected)	c.4429del p.(Leu1479Trpfs^∗^55)	Pathogenic	Paternal (affected)	[10]
4	Antenatal (TOP)	*c*.9499*A* > *T* p.(Ile3167Phe)	Paternal (unaffected)	*c*.5627*C* > *G* (p.Ser1876^∗^)	Pathogenic	Maternal (affected)	[14]
5a & b^∗^	Antenatal (neonatal demise × 2)	*c*.9499*A* > *T* p.(Ile3167Phe)	Maternal (unaffected)	*c*.2534 *T* > *C* p.(Leu845Ser)	Likely pathogenic	Paternal (affected)	This study
6a & b	Neonatal (kidney failure aged 17 and 22)	*c*.9499*A* > *T* p.(Ile3167Phe)	Assumed maternal (confirmed *in trans* in proband)	c.10071dup p.(Thr3358Hisfs^∗^32)	Pathogenic	Paternal (affected)	This study
7	Age 12 (kidney failure age 32)	*c*.9499*A* > *T* p.(Ile3167Phe)	Unknown	*c*.1198*C* > *T* p.(Arg400^∗^)	Pathogenic	Unknown	This study

^∗^No DNA available from 5b to confirm genotype; however, phenotype and obstetric history are consistent with VEO-PKD and 5a.

## Data Availability

The genetic data used to support the findings of this study are included within the article.

## Supplementary Materials

Supplementary 1 Supplementary methods. Supplementary Table 1: variant classification of PKD1 variants using ACMG 2015 & ACGS 2020 guidelines after bioinformatic filtering. Supplementary Table 2: summary run metrics for long-read Flongle datasets. Supplementary Table 3: phenotypic data at enrolment of individuals with the p.(Ile3167Phe) variant compared to other individuals in UKBB.

Supplementary 2 Supplementary Figure 1: MinION nanopore long-read sequencing performed on 100 K.1a and 1b to confirm phase showed that the two variants were inherited *in trans*.

## References

[1] Gilbert R. D., Sukhtankar P., Lachlan K., Fowler D. J. (2013). Bilineal inheritance of PKD1 abnormalities mimicking autosomal recessive polycystic disease. *Pediatric Nephrology*.

[2] Rossetti S., Kubly V. J., Consugar M. B. (2009). Incompletely penetrant *PKD1* alleles suggest a role for gene dosage in cyst initiation in polycystic kidney disease. *Kidney International*.

[3] Vujic M., Heyer C. M., Ars E. (2010). Incompletely penetrant PKD1 alleles mimic the renal manifestations of ARPKD. *Journal of the American Society of Nephrology*.

[4] Audrézet M.-P., Corbiere C., Lebbah S. (2016). Comprehensive PKD1 and PKD2 mutation analysis in prenatal autosomal dominant polycystic kidney disease. *Journal of the American Society of Nephrology*.

[5] Durkie M., Chong J., Valluru M. K., Harris P. C., Ong A. C. M. (2021). Biallelic inheritance of hypomorphic *PKD1* variants is highly prevalent in very early onset polycystic kidney disease. *Genetics in Medicine*.

[6] Losekoot M., Ruivenkamp C. A. L., Tholens A. P. (2012). Neonatal onset autosomal dominant polycystic kidney disease (ADPKD) in a patient homozygous for a PKD2 missense mutation due to uniparental disomy. *Journal of Medical Genetics*.

[7] Bergmann C., von Bothmer J., Ortiz Brüchle N. (2011). Mutations in multiple PKD genes may explain early and severe polycystic kidney disease. *Journal of the American Society of Nephrology*.

[8] Fowler A. (2022). DECoN: a detection and visualization tool for exonic copy number variants. *Methods in Molecular Biology*.

[9] Valluru M. K., Chung N. K. X., Gilchrist M. (2023). A founder *UMOD* variant is a common cause of hereditary nephropathy in the British population. *Journal of Medical Genetics*.

[10] Mantovani V., Bin S., Graziano C. (2020). Gene panel analysis in a large cohort of patients with autosomal dominant polycystic kidney disease allows the identification of 80 potentially causative novel variants and the characterization of a complex genetic architecture in a subset of families. *Frontiers in Genetics*.

[11] Hopp K., Ward C. J., Hommerding C. J. (2012). Functional polycystin-1 dosage governs autosomal dominant polycystic kidney disease severity. *The Journal of Clinical Investigation*.

[12] Ong A. C., Harris P. C. (2015). A polycystin-centric view of cyst formation and disease: the polycystins revisited. *Kidney International*.

[13] Xu Y., Streets A. J., Hounslow A. M. (2016). The polycystin-1, lipoxygenase, and *α*-toxin domain regulates polycystin-1 trafficking. *Journal of the American Society of Nephrology*.

[14] Janssens P., Decuypere J. P., de Rechter S. (2021). Enhanced MCP-1 release in early autosomal dominant polycystic kidney disease. *Kidney International Reports*.

[15] Senol-Cosar O., Schmidt R. J., Qian E. (2019). Considerations for clinical curation, classification, and reporting of low- penetrance and low effect size variants associated with disease risk. *Genetics in Medicine*.

