# Supplementary material for: The Common PKD1 p.(Ile3167Phe) Variant Is Hypomorphic and Associated with Very Early Onset, Biallelic Polycystic Kidney Disease
Source: Hum Mutat. 2023 Jul 28;2023:5597005. doi: 10.1155/2023/5597005 (PMC11918491; doi:10.1155/2023/5597005)
Supplement: Supplementary 1 — Supplementary methods. Supplementary Table 1: variant classification of PKD1 variants using ACMG 2015 & ACGS 2020 guidelines after bioinformatic filtering. Supplementary Table 2: summary run metrics for long-read Flongle datasets. Supplementary Table 3: phenotypic data at enrolment of individuals with the p.(Ile3167Phe) variant compared to other individuals in UKBB. [file 5597005.f1.docx]

**Supplementary methods**

**MinION long-read sequencing to determine phase**

Each reaction consisted of 2 μL of genomic DNA (40 ng/μL), 5 μL of 5× Long Amp Buffer, 1 μL of Long Amp Taq (2.5 U/μL), 1 μL of dNTPs, 15.25 μL of nuclease-free H_2_O and 2 μL of 10 pmol/μL forward (dCCGTGTAGAGAGGAGGGCGTGTGCAAGGA) and reverse (dTCGGCAAGGACCTGCTGGATCAGGTCTTC) primers. Thermocycling conditions were 94^o^C for 2 minutes, followed by 30 cycles at 94^o^C for 30 seconds, 69^o^C for 30 seconds, 65^o^C for 10 minutes and a final 65^o^C extension step for 10 minutes. The amplification products were purified using AMPure XP beads (Beckman Coulter, Indianapolis, Indiana, USA) in a 0.8× cleanup reaction which was eluted using 30 μL of buffer EB (Qiagen GmbH).

To create a Flongle-compatible sequencing library with kit LSK109 (ONT), an end-repair and nickase treatment reaction was first performed. The reaction comprised 1.75 μL formalin-fixed paraffin-embedded (FFPE) DNA repair buffer (New England Biolabs (NEB), Ipswich, Massachusetts, USA), 1 μL FFPE DNA repair mix, 1.75 μL Ultra^TM^ II end prep reaction buffer (NEB), 1.5 μL Ultra^TM^ II end prep enzyme mix (NEB) and 24 μL of 100 fmol PCR product. The reaction was incubated at 20^o^C for 5 minutes then 65^o^C for 5 minutes before being cleaned up using AMPure XP beads. Sequencing adapters were ligated to the double-stranded DNA in a reaction that comprised 30 μL of end-prep/FFPE treated PCR products, 12.5 μL LNB Ligation Buffer (ONT), 2.5 μL AMX Adaptor Mix (ONT), 5 μL Quick T4 Ligase (NEB). The reaction was incubated at room temperature for 10 minutes before a further AMPure cleanup was performed using Long Fragment Buffer (ONT) to wash the beads. The sample was eluted in 7 μL of buffer EB (ONT). To prime the Flongle flowcell 120 μL of flowcell priming mix (3 μL Flush Tether (ONT) combined with 117 μL Flush Buffer (ONT)) was loaded through the flowcell port. The prepared sample (15 μL SQB Sequencing Buffer (ONT), 10 μL LB Loading Beads (ONT) and 6 μL DNA library) was then immediately loaded. A 24-hour sequencing run was initiated using MinKNOW software v.4.4.3 (https://community.nanoporetech.com/downloads).

Offline basecalling was performed with Guppy v.6.1.5 (https://community.nanoporetech.com/downloads) using the super high accuracy (SUP) model, to convert the raw data from fast5 to FASTQ format. Adapter sequences were trimmed using Porechop v.0.2.3 (<https://github.com/rrwick/Porechop>) before NanoFilt v.2.2.0 (<https://github.com/wdecoster/nanofilt>) (De Coster, D'Hert, Schultz, Cruts, & Van Broeckhoven, 2018) was used to select reads based on their length (7003 to 8003 bp) and quality (≥10). Reads were then aligned to an indexed human reference genome (build GRCh38) using ngmlr v.0.2.7 (<https://github.com/philres/ngmlr>) (Sedlazeck et al., 2018). Parental haplotypes, defined by the single nucleotide variant at position chr16:2100465 enabled maternal and paternal reads to be selected using the Jvarkit tool biostar214299 (<http://lindenb.github.io/jvarkit/Biostar214299.html>) (Lindenbaum & Varkit, 2015). Samtools v.1.3.1 (http://www.htslib.org/) (Li et al., 2009) was used to aid file manipulations (SAM to BAM conversion, sorting reads by alignment coordinate and file indexing) and the Integrative Genome Viewer v.2.12.2 (<https://software.broadinstitute.org/software/igv/>) (Thorvaldsdottir, Robinson, & Mesirov, 2013) was used to visualise aligned sequence reads. NanoStat v.1.1.2 (https://github.com/wdecoster/nanostat) (De Coster et al., 2018) was used to generate assay performance metrics.

***Genomics England 100,000 Genomes Project***

Inclusion and genotyping of participants in the 100,000 Genomes Project (100K) was managed by Genomics England Limited (GEL). All participants in the 100K provided written consent to access their anonymized clinical and genomic data for research purposes (https://re.extge.co.uk/ovd/). Whole-genome sequencing (WGS) was performed on all participants. Phenotypes of identified carriers were manually reviewed in the Genomics England Participant Explorer.

**Supplementary Table 1: Variant classification of PKD1 variants using ACMG/ACGS guidelines after bioinformatic filtering.**

| **PKD1 variants detected** | **Evidence for/against pathogenicity** | **ACMG code & strength** | **Variant Classification** |
| --- | --- | --- | --- |
| c.2534T>C p.(Leu845Ser) | *De novo* in fetus with VEO PKD (Audrezet et al., 2016); Seen in multiple families with ADPKD (Audrezet et al., 2016; Rossetti et al., 2012; Thomas et al., 1999) including segregation data in one large family (Peltola et al.); 1/230128 alleles gnomAD; Phenotype consistent with ADPKD in multiple patients. | PS2_mod  PS4_mod  PP1_mod  PM2_sup  PP4_sup | Class 4 = likely pathogenic |
| c.9499A>T p.(Ile3167Phe) | 340 alleles including 2 homozygotes (0.21%) in gnomAD; Found with biallelic *PKD1* pathogenic variant in 4 previously reported unrelated VEO probands (Durkie et al., 2021; Janssens et al., 2021; Mantovani et al., 2020); REVEL 0.459; SIFT & Polyphen damaging. Protein modelling showed altered surface modelling affecting important PLAT domain. | BS1_str  BS2_str  PM3_str  PM1_sup | Likely hypomorphic variant* |
| c.11957C>T p.(Ala3986Val) | 36 alleles on gnomAD; REVEL 0.077 (Val in numerous mammals). Confirmed *in cis* with c.9499A>T in 2 local cases. | BS1_sup  BP4_sup | Class 2 = Likely benign |
| c.4681C>T p.(Pro1561Ser) | Not present in gnomAD; REVEL 0.456. Confirmed *in cis* with c.2534T>C p.(Leu845Ser) | PM2_mod  BP2_sup | Class 3 = Variant of uncertain significance |

All variants are described according to RefSeq PKD1: NM_001009944.3. Variant classification according to ACMG guidelines (Richards et al., 2015) and ACGS Best Practice Guidelines for Variant Classification in Rare Disease 2020. *Proposed classification by this study.

**Supplementary Table 2: Summary run metrics for long-read Flongle datasets.**

| **Case** | **Flowcell ID** | **Estimated run yield (Mb)** | **Raw read count** | **Length filtered read count*** | **Length and quality filtered read count^#^** | **Median Q score^** | **Read depth per haplotype** | |
| --- | --- | --- | --- | --- | --- | --- | --- | --- |
|  |  |  |  |  |  |  | g.2100465T | g.2100465A |
| 100K.1a | AEI784 | 175.41 | 33,102 | 14,839 | 8,237 | 11.8 | 4,066 | 3,435 |
| 100K.1b | AEQ200 | 367.99 | 57,626 | 30,915 | 17,450 | 12.2 | 8,684 | 7,253 |

*Retention of reads ≥7003 and ≤8003. ^#^Following retention of reads with a mean Q score ≥10. ^Of length and quality filtered reads. Genomic coordinates provided according to genome build Hg38.

**Supplementary Table 3: Phenotypic data at enrolment of individuals with the p.(Ile3167Phe) variant compared to other individuals in UKBB.**

|  | **PKD1 variant p.(Ile3167Phe)**  N=618* | **UKBB**  **controls**  N=450,375 | **P value** |
| --- | --- | --- | --- |
| **Age (years)** | 57.2±8.0 | 57.3±8.0 | 0.72 |
| **Sex (M/F%)** | 43.4/56.6 | 45.7/54.3 | 0.24 |
| **CKD-EPI eGFR (mL/min/1.73 m²)** | 92.4±12.7 | 90.5±13.0 | 0.0002 |
| **Systolic BP**  **(mm Hg)** | 144.4±23.4 | 144.2±24.1 | 0.64 |
| **Diastolic BP**  **(mm Hg)** | 86.5±13.6 | 86.4±13.5 | 0.82 |
| **ACR**  **(mg/mmol)** | 1.9±3.6 | 1.6±2.5 | 0.11 |

*Data for individuals of White European descent only

**Supplementary Figure 1:** MinION nanopore long-read sequencing, performed on samples 100K.1a and 1b to confirm phase, demonstrated that the two variants were inherited in *trans*.
