# Supplementary figures and images for: The Common PKD1 p.(Ile3167Phe) Variant Is Hypomorphic and Associated with Very Early Onset, Biallelic Polycystic Kidney Disease
Source: Hum Mutat. 2023 Jul 28;2023:5597005. doi: 10.1155/2023/5597005 (PMC11918491; doi:10.1155/2023/5597005)

Suppl Figure 1

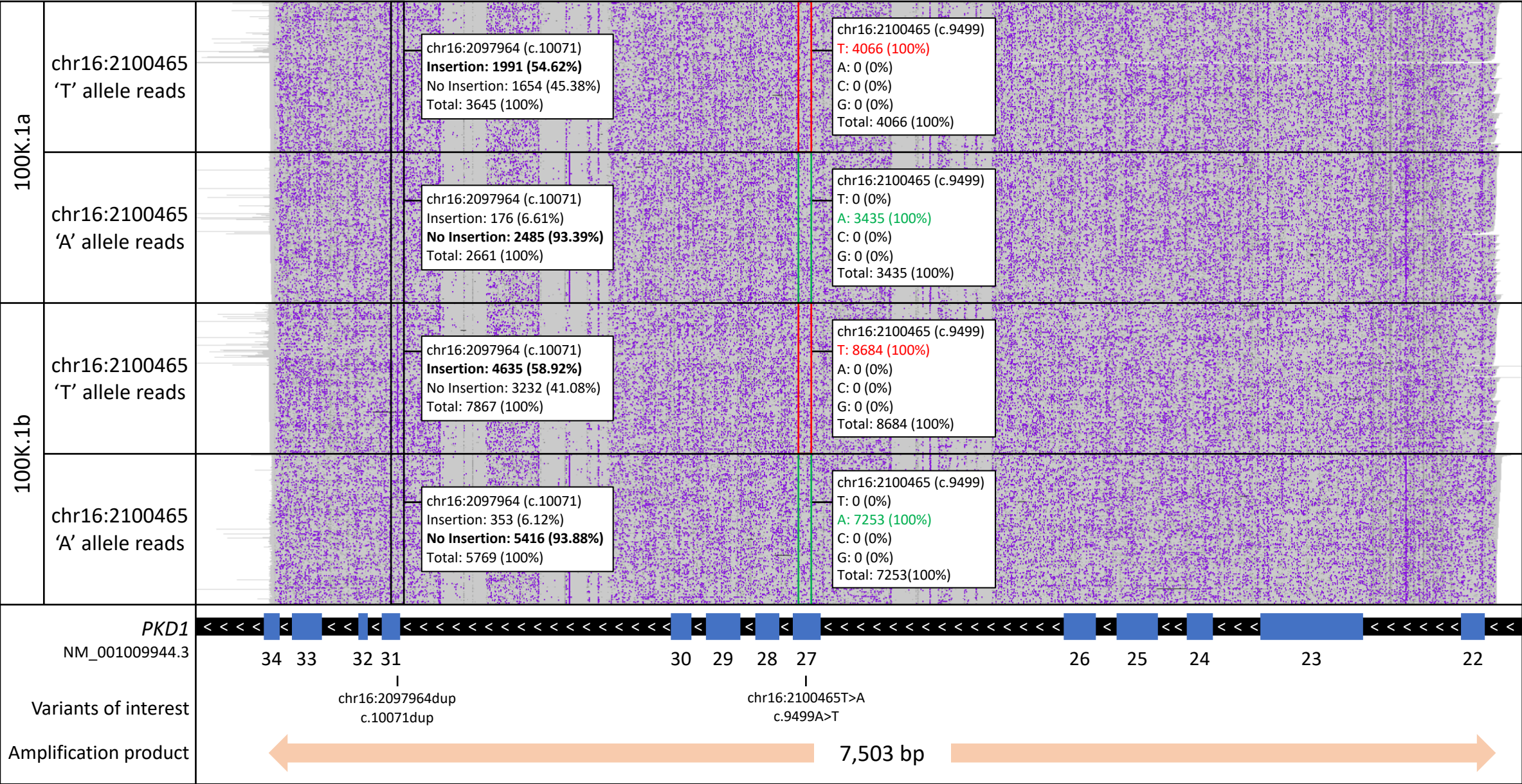

Supplement: Supplementary 2 — Supplementary Figure 1: MinION nanopore long-read sequencing performed on 100 K.1a and 1b to confirm phase showed that the two variants were inherited in trans. [file 5597005.f2.pdf]
